# Color Doppler evaluation of left gastric vein hemodynamics in cirrhosis with portal hypertension and its correlation with esophageal varices and variceal bleed

**DOI:** 10.4103/0971-3026.73541

**Published:** 2010-11

**Authors:** Subathra Adithan, Bhuvaneswari Venkatesan, Elangovan Sundarajan, Vikram Kate, Raja Kalayarasan

**Affiliations:** Department of Radiodiagnosis, Jawaharlal Institute of Postgraduate Medical Education and Research (JIPMER), Pondicherry - 605 006, India; 1 Department of Surgery, Jawaharlal Institute of Postgraduate Medical Education and Research (JIPMER), Pondicherry - 605 006, India

**Keywords:** Esophageal varices, grade of varices, hepatofugal flow velocity, left gastric vein

## Abstract

**Aim:**

The purpose of this study was to assess the value of Doppler evaluation of left gastric vein hemodynamics when monitoring portal hypertension patients, by correlating Doppler ultrasonography (USG) parameters with the severity of esophageal varices and occurrence of variceal bleeding.

**Methods:**

This study was carried out on 100 patients using Doppler USG and endoscopy. Forty-seven of these were patients with cirrhosis with portal hypertension, who had not had a recent variceal bleed (group 1) and 26 were patients with cirrhosis with portal hypertension, with a recent history of bleeding (group 2). The control group comprised of 27 subjects who did not have liver disease or varices on endoscopy (group 3). The hemodynamic parameters, namely the diameter of the left gastric vein and the direction and flow velocity in the vessel, were compared in these groups, with the grade of esophageal varices.

**Results:**

Hepatofugal flow velocity in the left gastric vein was higher in patients with large-sized varices compared to those patients with small-sized varices (*P* < 0.001). The left gastric vein hepatofugal flow velocity was higher in patients with a recent variceal bleed than in those patients without a history of a recent variceal bleed (*P* < 0.0149). Large-sized varices were more commonly found in patients with a history of a recent variceal bleed (*P* < 0.0124).

**Conclusion:**

Left gastric vein hemodynamics were found to correlate with the severity of the varices and the occurrence of recent variceal bleed in patients with cirrhosis with portal hypertension. Evaluation of the left gastric vein portal dynamics could be helpful in monitoring the progress of the disease in these patients.

## Introduction

Portal hypertension results in the development of spontaneous portosystemic collaterals at a number of anatomic sites as a response to increased pressure. The most clinically significant of these are the gastroesophageal varices because of their propensity to rupture and cause life-threatening massive hemorrhage. Gastroesophageal varices are supplied by an enlarged left gastric vein as well as short gastric veins arising from the splenic bed. The risk of variceal bleeding in a patient with portal hypertension is at present assessed by endoscopic grading of the varices and the presence of red signs on endoscopy. However, following endoscopic treatment of esophageal varices, such as banding and sclerotherapy, the varices get obliterated and it might become impossible to assess the status of the underlying portal hemodynamics by endoscopy. Schmassman *et al*. found that after sclerotherapy, the endoscopic findings showed no significant correlation with the prevalence of bleeding.[1] Besides, endoscopy can be expensive and uncomfortable and this can limit the frequency of examination.

Studies have shown variable associations between various Doppler parameters and portal hemodynamics. A study done in China, using CT portal venography, found a correlation between esophagogastric variceal bleeding and the location of the left gastric vein orifice on the portal system. They found a higher probability of esophagogastric variceal bleeding in patients in whom the left gastric vein was terminating in the portal vein than in patients in whom the left gastric vein was terminating in the splenic vein or splenoportal junction.[2] An earlier study done in 1991, had stated that variceal bleeding occurred more frequently in patients with a hepatopetal flow in the portal venous system (portal vein, splenic vein or superior mesenteric vein) than in patients with a hepatofugal flow, and that the hepatofugal flow in the left gastric vein need not be present for a variceal bleed to occur.[3] Later studies showed a correlation between the hepatofugal flow in the left gastric vein and variceal bleeding.[4,5] The left gastric vein velocity and diameter were found to correlate with the occurrence of variceal bleeding.[4] Others found that dilatation of the left gastric vein need not be present for variceal hemorrhage to occur.[6]

Blood flow velocity in the left gastric vein trunk and its branches and in the perforating veins might regulate the blood flow supplying the esophageal varices and contribute to their development. Hino *et al*. studied the left gastric vein hemodynamics using endoscopic *ultrasonography* (USG) and found that hepatofugal blood flow velocity in the left gastric vein trunk increased as the size of the varices increased, whereas, the diameter did not increase. They also found that the left gastric vein bifurcates into anterior and posterior branches, and as the size of the varices increased the branch pattern was more likely to be anterior branch dominant. There was no significant difference between the three grades of esophageal varices with respect to the size of the paraesophageal collaterals.[7]

To the best of our knowledge no similar study has been done in the Indian population. This study was undertaken to examine the potential benefits of Doppler USG in monitoring patients with portal hypertension.

## Materials and Methods

Prior approval was obtained from the ethics committee of our institute and a written informed consent was obtained from all patients. The patient characteristics are shown in Table 1.

**Table 1:** Patient characteristics

Characteristics	Group 1 (*n* = 47)	Group 2 (*n* = 26)	Group 3 (*n* = 27)
Sex			
Male	24 (51.1%)	16 (61.5%)	15 (55.6%)
Female	23 (48.9%)	10 (38.5%)	12 (44.4%)
Age (years) mean ± SD	38.2 ± 14.4	39.5 ± 12.7	42.9 ± 19.75
Diagnosis	Cirrhosis without recent variceal bleed	Cirrhosis with recent variceal bleed	Patients without liver disease or portal hypertension
Prior treatment	Sclerotherapy (11); banding (0); both sclerotherapy and banding (5); no treatment (31)	Sclerotherapy (10); banding (4); both sclerotherapy and banding (0); no treatment (12)	Not applicable

Patients taking part in the study were referred from the surgery and medicine endoscopy rooms. A total of 100 patients were studied by Doppler USG. The diagnosis of cirrhosis was made based on clinical and laboratory findings. The cases were categorized into three groups.

Group 1 (*n* = 47): Those who were diagnosed to have cirrhosis with portal hypertension and did not have a history of variceal bleed during the preceding four weeks

Group 2 (*n* = 26): Those who were diagnosed to have cirrhosis with portal hypertension and had a history of variceal bleed during the preceding four weeks

Group 3 (*n* = 27): Those who did not have any features of liver disease or portal hypertension and had normal endoscopic findings

The duration of disease and treatment history was noted in all the participants. Ultrasound examination was done on the participants of the study using a standard color Doppler USG scanner (ECOCEE, Toshiba, Otawara, Japan) with a 5-MHz convex probe. All the participants were examined after overnight fasting and during quiet respiration.

The endoscopic examination was completed on all the patients (with cirrhosis and portal hypertension) taking part in the study within one week of the Doppler examination during which the grade of the esophageal varices was noted. Subjects who had no evidence of liver disease, in whom endoscopy was done for other indications such as dyspepsia or suspected acid peptic disease and in whom the endoscopy findings were normal, were taken as controls. The Doppler examination was completed on these subjects (controls) within one week of the endoscopic examination.

Esophageal varices were graded as follows[8]:

Grade 1: Visible veins, not elevated

Grade 2: Raised large veins, not touching one another

Grade 3: Raised large tortuous veins, almost touching one another

Grade 4: Very large dilated veins, filling the esophageal lumen

Patients in whom no varices were seen or in whom varices were obliterated following endoscopic sclerotherapy or banding were assumed to have grade 0 varices. Patients with variceal grades 1 and 2 and patients whose varices were obliterated as a result of previous endoscopic banding or sclerotherapy were grouped together as having small-sized varices. Patients with variceal grades 3 and 4 were considered to have large-sized varices.

The examination was performed in the supine, right oblique, and left oblique positions. The wall filter was kept at 100 Hz.

**Figure 1:**
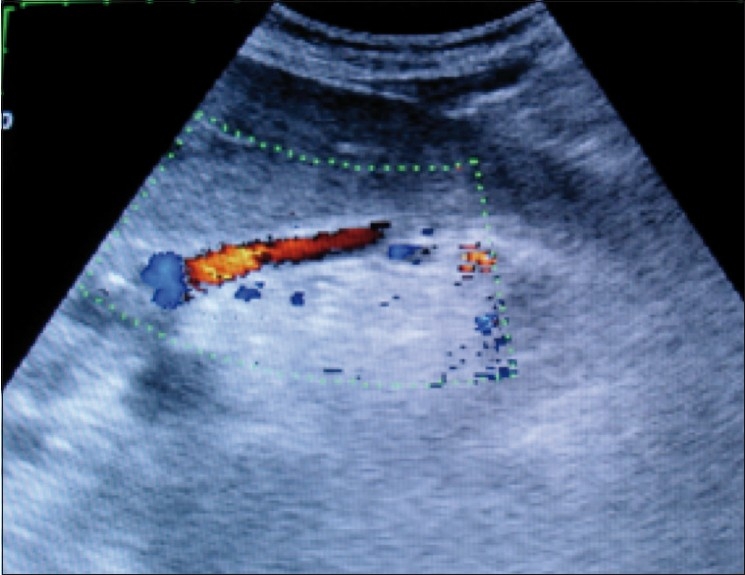
Color Doppler USG shows a dilated left gastric vein showing hepatofugal flow in a patient with portal hypertension

The left gastric vein was identified as a cephalad-directed vessel arising from the portal vein, portal vein confluence, or the splenic vein going toward the gastroesophageal junction [Figure 1]. The diameter, flow direction, and maximum flow velocity in the left gastric vein were noted.

### Statistical analysis

The groups with and without recent variceal bleed were compared and analyzed for differences in the measured Doppler USG parameters. The parameters were also compared with the grade of varices. Statistical analysis was done using a standard software (SPSS for Windows, version 13, Chicago, USA). The unpaired *t* test was used for comparing two unpaired groups, one-way analysis of variance (ANOVA) was used for multiple comparisons, the chi-square test for comparing categorical variables, the Mann–Whitney was used for nonparametric analysis, and the Fisher’s exact test was used for contingency tables. A *P* value less than 0.05 was considered to be significant.

## Results

Table 1 shows the patient characteristics. Table 2 shows the grade of varices. Large-sized varices were more commonly found in patients with a history of a recent variceal bleed (*P* < .0124).

**Table 2:** Grade of varices by endoscopy

Parameters		Group 1 (*n* = 47)	Group 2 (*n* = 26)
Small-sized varices	0	11 (23.4%)†	0
	1	18 (38.3%)	5 (19.2%)
	2	11 (23.4%)	10 (38.5%)
Large-sized varices	3	4 (8.5%)	7 (26.9%)
	4	3 (6.4%)	4 (15.4%)
Total		47 (100%)	26 (100%)

Groups 1 versus 2, small-sized varices versus large-sized varices, *P* < 0.0124 (Fisher’s exact test)

**Table 3:** Ultrasound parameters of the left gastric vein (values are mean ± SD)

	Group 1 (*n* = 47)	Group 2 (*n* = 26)	Group 3
Visualization	14 (29.8%)	14 (53.8%)*	0
Mean diameter (mm)	4.92 ± 2.3	5.43 ± 2.8	-
Number of patients with hepatopetal flow	9	8	-
Number of patients with hepatofugal flow	5	6	-
Mean hepatopetal flow velocity (cm/s)	14.8 ± 7.9	12.9 ± 9	-
Mean hepatofugal flow velocity (cm/s)	15.4 ± 12.1	37 ± 11.6†	-

* *P* < 0.0495;

† *P* < 0.0149 for group 1 versus 2. Values are mean ± SD

The left gastric vein was not adequately visualized in any of the patients of the control group. It was more frequently visualized in patients with a recent variceal bleed than in patients without a recent variceal bleed (*P* < 0.045). The left gastric vein could be visualized in 29.8% of those without a history of recent variceal bleed and in 53.85% of those with a history of a recent variceal bleed. In this study, the left gastric vein was visualized in 38.4% of the patients with portal hypertension. The findings related to the left gastric vein in groups 1 and 2 are shown in Table 3.

The diameter and flow direction did not significantly differ between the two groups. The mean diameters associated with different grades of varices are shown in Table 4. The flow direction associated with the different grades of varices is shown in Table 5.

**Table 4:** Mean diameter of the left gastric vein in the two groups in different grades of varices

Grade of varices		Group 1 (mm)	Group 2 (mm)
Small-sized varices	0	4.4	-
	1	4.34	4.6
	2	3.6	5.1
Large-sized varices	3	8	6.6
	4	11	5

Groups 1 versus 2 (*P* = ns)

**Table 5:** Direction of the flow of the left gastric vein in the two groups in different grades of varices

Grade of varices		Direction of flow	Number of patients	
			Group 1	Group 2
Small-sized varices	0	Hepatopetal	3	
		Hepatofugal	1	-
	1	Hepatopetal	2	2
		Hepatofugal	3	-
	2	Hepatopetal	2	3
		Hepatofugal	1	1
Large-sized varices	3	Hepatopetal	-	-
		Hepatofugal	1	4
	4	Hepatopetal	1	3
		Hepatofugal	-	1

A diameter greater than 6 mm was more frequent in patients who had a recent variceal bleed (58%) than in those who did not have a recent variceal bleed (12%); however, this difference was not statistically significant.

The hepatofugal flow velocity was higher in the left gastric vein in large-sized varices (grade 3 and 4) compared to the flow velocity in small-sized varices (grades 1 and 2 and obliterated varices) (*P* < .001). The hepatofugal flow velocity in the different grades of varices is shown in Table 6.

**Table 6:** Mean hepatofugal left gastric vein (LGV) velocity in different grades of varices

Grade of varices		Mean hepatofugal LGV velocity (cm/s)
	0	10
Small-sized varices	1	9.5
	2	15
Large-sized varices	3	41.2
	4	34

Small-sized varices versus large-sized varices, *P* < 0.001

The mean hepatopetal flow velocity was 14.8 ± 7.9 cm/s in patients without a recent variceal bleed and 12.9 ± 9.01 cm/s in patients with a recent variceal bleed. The mean hepatofugal flow velocity was 15.4 ± 12.1 cm/s in patients without a recent variceal bleed and 37 ± 11.6 cm/s in patients with a recent variceal bleed. The mean hepatofugal flow velocity was higher in patients with a recent variceal bleed (*P* < 0.0149) [Table 3].

## Discussion

In our study, in the group without a recent variceal bleed, 34% had undergone some kind of endoscopic treatment (sclerotherapy or banding) for varices. In the group with a history of recent variceal bleed, 54% had undergone some kind of endoscopic treatment for varices before the Doppler examination was performed on them. We assumed that the current grade of varices would reflect the underlying Doppler hemodynamics at that moment, and that a past history of endoscopic treatment might not significantly interfere in this relationship.

The hepatofugal flow in one of the main branches of the portal venous system (portal vein, splenic vein or superior mesenteric vein) was found in 6.8% of the subjects in our study, which was similar to the finding obtained in other studies.[3,9]

The hepatofugal flow in large collateral vessels, creating a portosystemic shunt, is associated with a reduced incidence of bleeding;[3] however, hepatofugal flow in the coronary vein is associated with a higher risk of variceal bleeding. In this study, paraumbilical veins were seen in three patients who had not bled, but in none of the patients who had bled. This might be because of the protective effect paraumbilical varices exert by diverting blood away from the gastroesophageal junction. Splenorenal varices were seen in three patients who had not bled, but only in one patient who had bled recently. This difference did not reach statistical significance due to the small numbers involved, but splenorenal varices also exert a protective effect against the variceal bleed by diverting blood away from the gastroesophageal junction. Extensive collateralization in other regions decompresses the portal venous system and diverts blood away from the gastroesophageal junction, resulting in a lower incidence of variceal bleed in these patients.

A limitation to the widespread application of LGV Doppler in the evaluation of patients with portal hypertension, is because the left gastric vein may not be adequately visualized by transabdominal USG, especially in patients with cirrhosis, but with no history of variceal bleed. In this study the left gastric vein was more commonly seen in patients who had had a recent variceal bleed (53.8%) [Table 3] than in patients who did not have a recent variceal bleed (29.8%) (*P* < 0.045). When adequately visualized, the left gastric vein could be used in monitoring patients with portal hypertension.

The hepatofugal flow velocity in the left gastric vein was higher in patients who had had a recent variceal bleed. The hepatofugal flow velocity also shows a statistical correlation with the grade of esophageal varices. Li *et al*. reported similar results.[4] In another study, Kuramochi *et al*, also showed that LGV hemodynamics can predict the early recurrence of esophageal varices, after eradication of varices by endoscopic sclerotherapy or endoscopic variceal ligation.[10]

In conclusion, the left gastric vein hemodynamics show a correlation with the size of the varices and the occurrence of variceal bleed. Its value in predicting the occurrence of variceal bleed can be better established with larger prospective studies. The Doppler study of the left gastric vein may be particularly useful in the follow-up of patients who have already been treated with sclerotherapy or banding.
